# A Cognitive-Perceptual Approach to Conceptualizing Speech Intelligibility Deficits and Remediation Practice in Hypokinetic Dysarthria

**DOI:** 10.4061/2011/150962

**Published:** 2011-09-12

**Authors:** Kaitlin L. Lansford, Julie M. Liss, John N. Caviness, Rene L. Utianski

**Affiliations:** ^1^Motor Speech Disorders Laboratory, Department of Speech and Hearing Science, Arizona State University, P.O. Box 870102, Tempe, AZ 85287-0102, USA; ^2^Mayo Clinic, 13400 East Shea Boulevard, Scottsdale, AZ 85259-5452, USA

## Abstract

Hypokinetic dysarthria is a common manifestation of Parkinson's disease, which negatively influences quality of life. Behavioral techniques that aim to improve speech intelligibility constitute the bulk of intervention strategies for this population, as the dysarthria does not often respond vigorously to medical interventions. Although several case and group studies generally support the efficacy of behavioral treatment, much work remains to establish a rigorous evidence base. This absence of definitive research leaves both the speech-language pathologist and referring physician with the task of determining the feasibility and nature of therapy for intelligibility remediation in PD. The purpose of this paper is to introduce a novel framework for medical practitioners in which to conceptualize and justify potential targets for speech remediation. The most commonly targeted deficits (e.g., speaking rate and vocal loudness) can be supported by this approach, as well as underutilized and novel treatment targets that aim at the listener's perceptual skills.

## 1. Introduction

Hypokinetic dysarthria, a common manifestation of Parkinson's disease (PD), affects roughly 90% of the patient population [1, 2]. Moreover, hypokinetic dysarthria is a prominent feature of more severe and medically refractory parkinsonian disorders (e.g., progressive supranuclear palsy, multiple systems atrophy). Hypokinetic dysarthria is characterized perceptually by varying degrees of reduced pitch variation (monotonicity), reduced loudness, breathy voice, imprecise consonants, variable speaking rate, and short rushes of speech [1, 3, 4]. Reduced intelligibility occurs when these abnormal speech features interfere with the listener's ability to understand the spoken message. Intelligibility deficits can significantly reduce quality of life, contribute to depression and feelings of isolation, and hinder the ability to maintain gainful employment [5, 6]. Unlike trunk and limb motor impairments in PD, speech deficits typically do not respond vigorously to pharmacological or surgical interventions (see [7, 8] for reviews of the literature). Thus behavioral treatments to improve speech intelligibility constitute the bulk of speech treatment for this population.

Behavioral interventions by speech-language pathologists primarily aim to reduce or compensate for the underlying speech deficits to improve speech intelligibility [1]. Despite a growing body literature that generally supports the efficacy of various interventions, much work remains to establish a rigorous evidence base [9–11]. Further, there is no validated gold standard for treatment of intelligibility deficits in hypokinetic dysarthria because alternative interventions have not been systematically investigated [9, 10]. This absence of definitive research leaves both the speech-language pathologist and referring physician with the task of determining feasibility of clinical intervention for speech intelligibility remediation in PD. 

The purpose of this paper is to introduce a novel framework for medical practitioners in which to conceptualize and justify potential targets for speech remediation. Toward this end, we first address the construct of speech intelligibility and unique challenges hypokinetic speech pose to listeners. We then describe a cognitive-perceptual approach to conceptualizing potential treatment targets for improving intelligibility and, importantly, the mechanisms by which this improvement is achieved. Ultimately this paper will demonstrate how treatment targets can be justified using a theoretical approach, particularly in the absence of a rigorous evidence base. This approach accommodates the most commonly targeted deficits (rapid speaking rate and reduced vocal loudness), as well as underutilized and novel treatment targets that aim exclusively at the listener's perceptual skills.

## 2. Theoretical Models of Speech Perception

In its simplest terms, intelligible speech is that which can be understood by the listener. A time-varying acoustic signal (i.e., speech) activates the series of words that have been spoken in the listener's mental lexicon (see [12] for a review of models of speech perception and word recognition). Under optimal circumstances in daily living, this process proceeds automatically and with high levels of accuracy. However, the process by which this occurs is infinitely more complex than the simple mapping of a stream of acoustic information onto the words stored in the lexicon. Numerous variables interact synergistically in speech perception: the quality of the acoustic speech signal, the type and amount of noise in the environment, the listener's familiarity with the speaker and knowledge of the topic being discussed, and even the presence and quality of visual/facial movements of the speaker (e.g., see [13–18]). By synergistically, we mean that one cue may compensate for the degradation of another. For example, some degree of acoustic speech degradation may be offset by the listener's expectations about the message by applying top-down knowledge [16]. Similarly speech intelligibility may be facilitated by viewing concomitant speaker mouth movements [17]. This flexibility of cue use by listeners accounts for much of the ease with which speech is accurately and easily perceived. However, the complexity of the process is illustrated when degradation of any of these variables sufficiently interferes with the listener's ability to automatically recognize the spoken words [18]. This then requires the listener to apply more cognitive-perceptual resources to the task of deciphering speech, invoking higher order decision-making [16].

When looking to theories of speech perception, “trying to understand what is being said” can be reduced to the basic cognitive-perceptual processes that are invoked in response to degraded speech [12]. The fundamental task upon encountering an unintelligible utterance is *lexical segmentation*, the process of making decisions about where one word ends and another begins [19]. Lexical segmentation of nondegraded speech occurs automatically through the process of word recognition, such that listeners have the experience of hearing a string of spoken words as someone speaks [20]. However, when speech is substantially degraded, word boundaries are less apparent, similar to the experience of listening to someone speak an unfamiliar foreign language. In such instances, active effort for lexical segmentation is necessary. Mattys and colleagues [20] developed a hierarchical model that hypothesizes the circumstances associated with active lexical segmentation. When higher-level lexical, contextual, and phonemic information cannot be used to identify word boundaries, listeners rely on a number of lower-level acoustic cues to make these determinations, such as the likelihood of certain sounds occurring together in sequence, speech rhythm, and changes in pitch. Since the majority of word onsets in the English language are strong syllables [19], the task of lexical segmentation of degraded speech is facilitated by the presence of acoustic cues to syllabic stress [19–24]. This is known as the *metrical segmentation strategy. *Listeners also use signal-complementary information (see [25]), such as their knowledge of syntax, semantics, and even the topic about which the speech is centered to parse the speech signal. This signal-complementary information is very useful for priming potential lexical candidates. For example, if a listener understands one word and it is a noun, verbs related to that noun are primed [26, 27]. This combination of bottom-up and top-down decision-making is used to determine word boundaries when the acoustic information is so degraded as to prohibit automatic recognition of the string of words [25]. Once the speech stream is parsed, the acoustic information within each of the word-sized frames activates similar words in the listener's mental lexicon. This cognitive-perceptual process of *lexical activation *identifies a cohort of possible word candidates that the word-sized packet of acoustic information may represent. The listener considers this cohort and the degraded acoustic input and that which best matches the mental representations results in recognition of the spoken words. 

Success in lexical segmentation and lexical activation is contingent on the presence and quality of salient acoustic cues. This is especially true when the listener has limited knowledge regarding the speaker's message (i.e., when top-down knowledge is of limited use in the decision process) [20]. While it is beyond the intent of this paper to detail acoustic cue degradation, a few words are warranted regarding levels of analysis. At the “*phoneme*” level, speech sounds can be distorted (e.g., “ship” for “sip”), omitted (e.g., “sop” for “stop”), or substituted with another phoneme (e.g., “tip” for “sip”). The “*prosodic*” elements of the speech (rate, rhythm, and melody) also may be abnormal in variation or magnitude. The patterns of decrement across levels determine their impact on successful lexical segmentation and activation (see [28] for a more detailed discussion). Thus, intelligibility is compromised when (1) the listener incorrectly parses the speech signal, thereby activating a cohort that does not contain the intended word or (2) the signal is segmented correctly, but the degraded acoustic information within the segment prevents the selection of the intended word. This approach allows us to further understand the nature of intelligibility breakdowns.

### 2.1. Intelligibility of Hypokinetic Dysarthria

When hypokinetic dysarthria is sufficiently severe to reduce intelligibility, the processes of lexical segmentation and lexical activation may be challenged in ways that require increased cognitive effort by the listener and ultimately impact communicative success. Although hypokinetic dysarthria varies in presentation and severity across patients, there are speech features that are commonly exhibited. Thus, it can be hypothesized, along with some support from the literature, that degradation of certain acoustic cues or constellations of cues will have a predictable impact on lexical segmentation and activation. For example, phonemic uncertainty may be introduced by (1) articulatory imprecision resulting in speech sound distortions and/or (2) poor audibility of the speech signal (due to weak/breathy phonation). Such phonemic uncertainty may hinder the listener's ability to use lexically guided speech segmentation strategies and may also impede accurate activation of the target. The prosodic features of hypokinetic speech (e.g., accelerated and/or variable speaking rate, short rushes of speech, dysfluency, monopitch and monoloudness) may result in reduced cues to syllabic stress. Recall that syllabic stress cues become important for identifying word boundaries, particularly when the acoustic-phonetic information is degraded, thereby prohibiting lexically guided speech segmentation.

Lexical segmentation of hypokinetic dysarthric speech in PD has been the focus of a series of studies [24, 29–31] that have illustrated the perceptual challenges posed by these constellations of speech abnormalities. Liss et al. [29, 30] found that listeners were generally able to use the available acoustic cues in moderate to severe hypokinetic dysarthria to identify word boundaries. The listener error patterns revealed a significant tendency to treat strong syllables as word onsets, as is predicted by the metrical segmentation strategy hypothesis [22]. However, this tendency was less robust than for normal speech presented at low listening levels [21]. This provides support for the interpretation that part of the intelligibility reduction in hypokinetic dysarthria is linked to the reduced acoustic-perceptual constrastivity between strong and weak syllables. 

## 3. Cognitive-Perceptual Approach To Conceptualizing Speech Remediation Practice of Hypokinetic Dysarthria

The previous section defined the theoretically relevant cognitive-perceptual processes that must be undertaken to successfully decipher degraded speech. It is not our intent to suggest veridicality of these processes, nor that these processes comprise the correct or only conceptualization for intelligibility. Instead, the approach—one of joint and bi-directional consideration of the quality of the acoustic signal and the ways in which listeners process that signal—is the key element of the model. Previous studies have provided a foundation of support for this approach (e.g., [16, 24, 28–32]). This approach begs the following question at every decision point in clinical practice: which modifications to the speech signal and/or listener will be most robust to facilitate the cognitive-perceptual processes of lexical segmentation and activation? In this way, the decision process is driven by the specific mechanisms by which intelligibility is enhanced. In contrast, the vast majority of behavioral interventions for hypokinetic dysarthria are aimed at reducing the deviant speech features of excessive articulation rate and/or increasing vocal loudness. The following will summarize current clinical practice and demonstrate how this approach can be used to justify these interventions as a rigorous evidence base is being developed. We also will demonstrate the utility of this approach for existing and novel signal-complementary interventions.

### 3.1. Rate Reduction Techniques

#### 3.1.1. Current Practice

Hypokinetic dysarthric speech rate often is judged to be excessive. Despite preserved velocity of movement, range is restricted, and this, along with articulatory imprecision, is thought to give the impression of rapid, mumbled speech [33, 34]. Rate control intervention strategies are additionally motivated by findings that suggest that some dysarthric speakers are more intelligible when they slow their rate of speech [35–37]. The use of rate reduction techniques to treat hypokinetic dysarthria is supported by kinematic evidence that suggests labial movements are restricted at habitual speaking rates but approximate those of healthy controls at slower rates [33]. Thus, rate reduction techniques may serve to improve articulatory precision. 

A variety of methods have been developed to reduce the speaking rate of dysarthric patients (for a comprehensive review of these methods see [38]). Generally, these techniques fall into one of two categories: (1) those that impose a metered or “rigid” speaking rate or (2) those that preserve natural speaking rhythm (i.e., global rate control techniques). Rigid rate control techniques impose a one word- or syllable-at-a-time speaking style and utilize such tools as pacing boards and alphabet supplementation boards. These methods have been criticized as being disruptive to natural speech rhythm [37], which is an important cue for lexical segmentation. Global rate control methods, such as the use of specific cueing/pacing strategies or delayed/altered auditory feedback, have the potential to preserve natural speech rhythm.

Despite the variety of methods developed to reduce speaking rate, efficacy of these techniques to improve intelligibility in hypokinetic dysarthria remains to be established. To date, three systematic reviews of speech treatment literature for dysarthria have been published [9–11]. In both the Cochrane [9] and American Academy of Neurology (AAN) reviews [10], no studies of rate reduction intervention met the inclusionary criteria and were therefore not evaluated for efficacy. The systematic review of loudness, rate, and prosody treatments in dysarthria published by the Academy of Neurologic Communication Disorders and Sciences (ANCDS) identified seven studies that evaluated the effects of rate reduction techniques in the treatment of hypokinetic dysarthria [11]. This review concluded that rate reduction techniques may facilitate intelligibility, but their success appears contingent on a number of conditions that must be scientifically addressed (e.g., type of dysarthria being treated, method of rate reduction used). Results from recent investigations not considered by these systematic reviews offer some support for the use of rate reduction techniques in the treatment of hypokinetic dysarthria, but do not point to any one method as being most effective [39–41].

#### 3.1.2. Cognitive-Perceptual Approach

How might rate reduction techniques facilitate the cognitive-perceptual processes of lexical segmentation and activation? Consider what is affected with the implementation of speech rate reductions. Speaking slowly has been shown to improve phonemic distinctiveness (i.e., articulatory precision) in healthy control speakers [42]. Likewise, Tjaden and Wilding [43] found that speaking slowly expanded the vowel working space in patients with ataxic dysarthria secondary to multiple sclerosis (although this finding was not found for their speakers with hypokinetic dysarthria secondary to PD). Such phonemic distinctiveness has been demonstrated to be a predictor of intelligibility in the speech of controls [44] and people with dysarthria [45–47]. 

In our approach, improved articulatory precision serves to decrease phonemic uncertainty. Recall that listeners only resort to lower-level cues, such as prosody, to guide lexical segmentation when higher-level cues to phonemic identity are ambiguous [20]. Therefore, reduced phonemic ambiguity would facilitate the listener hearing a “string of words” and automatically segment by way of word recognition. Even if phonemic ambiguity remains, the improved vowel space observed with slowing would facilitate the use of prosody and rhythm to distinguish strong and weak syllables, thereby allowing a metrical segmentation strategy by the listener. Further, the task of lexical segmentation should be facilitated by global rate reduction techniques, which aim to preserve natural speech rhythm. In rigid rate reduction techniques (e.g., pacing), the task of lexical segmentation is unnecessary because pauses are placed at word boundaries, so the listener's task would be restricted to matching the intervening acoustic information with the activated word representation. In addition, by slowing the rate of speech, listeners are afforded additional processing time to segment the speech signal and resolve the contents of these segments, via lexical segmentation and activation respectively, thus, providing them a better chance of deciphering the speaker's message.

### 3.2. Increased Loudness Techniques

#### 3.2.1. Current Practice

Reduced loudness (i.e., hypophonia) is a common manifestation of hypokinetic dysarthria and is considered to be a primary contributor of the resultant intelligibility disorder. Reduced vital lung capacity, chest wall rigidity, and glottal incompetence are a few examples of the physiological presentations of respiratory and phonatory insufficiency, which is the presumed cause of hypophonia observed in the PD population [48–50]. These physiological findings largely have been attributed to the overall muscle rigidity caused by PD [1, 3, 4]. Recently, however, abnormal neural drive to speech musculature and abnormal sensorimotor gating, rather than muscle rigidity, has been hypothesized to cause the respiratory/phonatory insufficiency observed in patients with PD [51, 52]. Working within this framework of causation, it is impairment of the use of internal cues that results in diminished speech movement initiation, amplitude, and timing [51, 53]. This suggestion is supported by electromyographic (EMG) findings (e.g., [54]) and is cited as the theoretical base for the use of behavioral techniques that aim to increase vocal loudness by providing external cues [51]. Some evidence suggests that provision of external cues to rescale the amplitude of movement, whether it is for speech or limb movements, temporarily ameliorates such conditions as hypophonia and micrographia in patients with PD [55, 56]. 

Lee Silverman Voice Treatment (LSVT; also known as LSVT/LOUD), a behavioral technique that elicits louder speech by providing external cues, is unparalleled in its popularity and widespread use. LSVT is described as a program that “delivers treatment in an intensive dose (60-minute individual sessions 4 days/week for 4 weeks), with multiple repetitions of each (speech) task (e.g., minimum of 15 repetitions per task per day), and continually increases requirements for effort, consistency and accuracy of vocal loudness in speech tasks” ([57, page 289]). LSVT is a registered trademark and can only be used by clinicians who successfully complete requisite workshops, which are now offered worldwide [58]. LSVT trained clinicians are permitted to use efficacy data to market and support reimbursement of LSVT [59]. LSVT uses the terms “clinically proven” and “level one evidence” on their website and in brochures to characterize their efficacy data [60].

However, as with rate reduction, efficacy research on LSVT does not yet have a rigorous evidence base. The Cochrane analysis [9] identified one LSVT study that qualified as randomized control trial (RCT; [61]), but with methodology rated as poor. The AAN review [10] identified two Class II studies (RCTs) that investigated the effects of LSVT on speech outcome measures that were assigned Level C evidence, indicating that LSVT *may* be considered to improve speech volume (i.e., loudness) [62, 63]. The ANCDS review [10] identified 16 Phase I, II, or III studies that described the effects of LSVT or cued loudness in hypokinetic dysarthria and concluded that LSVT produced significant improvements in vocal loudness in patients with PD. However, none of these studies included objective perceptual outcome measures that capture intelligibility (e.g., percent words correct from a transcription task, scaled intelligibility estimates in which the raters were blinded to the treatment condition). While evidence supporting improvements to intelligibility is emerging from case studies [64, 65], well-designed RCT studies are needed to validate the effectiveness of LSVT in improving speech intelligibility in hypokinetic dysarthria.

#### 3.2.2. Cognitive-Perceptual Approach

Despite the lack of a rigorous evidence base, the theoretical motivation underlying LSVT is supported. For example, listeners of hypokinetic speech that is produced louder enjoy greater intelligibility benefits than listeners of digitally amplified hypokinetic speech [66]. This is likely due to the acoustic changes associated with producing louder speech. Loud speech has been demonstrated to increase vocal intensity, improve use of pitch [66, 67], change vowel formant values and ratios [68], and alter articulatory displacements [69, 70]. How would these acoustic changes promote cognitive-linguistic processing of the speech? Unlike rigid rate reduction techniques, speaking loudly does not result in the presence of obvious word boundaries. However, these acoustic changes have the potential to improve use of cues for the task of lexical segmentation. The production of loud speech, therefore, not only improves overall audibility of the speech signal but also improves production of syllabic stress cues (e.g., pitch, vowel production). While relatively preserved, acoustic cues to syllabic stress in hypokinetic dysarthria are reduced [29]. Thus, treatments that aim to improve the contrast between stressed and unstressed syllables should promote lexical segmentation in hypokinetic dysarthria. The use of stress cues to achieve accurate lexical segmentation will permit an increased chance of the intended target to be activated. The other acoustic/articulatory changes associated with loud speech, such as increases in vowel space area and articulatory displacements that approximate those of healthy control speakers, result in greater articulatory precision. Thus greater articulatory precision reduces phonemic uncertainty and cognitive load, thereby facilitating lexical activation and word recognition.

### 3.3. Modifying Signal-Complementary Information

#### 3.3.1. Current Practice

The idea of modifying signal-complementary information or augmenting the listener's task of deciphering what has been said, has been commonplace in speech intelligibility practice [71–75]. By its very nature, targeting the listener rather than the speaker is often used when there is little opportunity for the speaker to improve their speech output. Augmentative communication strategies are typically reserved for the most severely affected individuals and as a last resort to continue oral communication. 

Signal-complementary information is that which is extraneous to the speech signal but has the potential to facilitate the understanding of what has been said. Techniques include the use of topic cues, alphabet cues, gestures, and formulation of predictable utterances (see [76] for a detailed account of these techniques). For example, Hustad and her colleagues [77] have reported alphabet cuing to improve listener performance, and sentence and word-level improvements in intelligibility have been reported when listeners were presented with semantically related cues [72, 78] or sentence topic [74].

Although the benefit of enhanced signal-complementary information to improved intelligibility is intuitively obvious (e.g., pointing to the first letter of a word using an alphabet board will facilitate word recognition), the variety of techniques and their relative effectiveness have not been formally evaluated. There is substantial and growing body of evidence that the utility of any given technique will depend on the type and severity of the speech degradation [73, 79] and on the characteristics of the listener (in particular, older versus younger; [74, 80–82]). As is the case with the traditional behavioral techniques that target remediation of the speech signal, the evidence base for the effectiveness of signal-complementary information to improve intelligibility is lacking. However, these techniques show promise in the treatment of the intelligibility decrements caused by dysarthria and have the added benefit of improved communication when the speaker is unable to improve their speech.

#### 3.3.2. Cognitive-Perceptual Approach

It is perhaps the obvious benefit afforded by signal-complementary approaches (i.e., approaches that do not focus on altering the speech signal) that has not warranted their assessment within a theoretical framework. However we suggest that doing so will allow for the systematic investigation of determining the most appropriate and effective intervention target for a given type of listener or listeners.

**Delimiting the Lexical Pool**

There are several commonly used signal-complementary strategies designed to facilitate word recognition. Using the approach offered here, the mechanisms can be defined as delimiting the pool of lexically activated items by (1) priming, or lowering the threshold for activation, of relevant words and (2) raising the threshold of activation for nonrelevant words. For example, alphabet cuing restricts lexical activation to the cohort of words that begin with a given letter and topic cuing causes relevant words to have a lower activation threshold than nonrelevant words. By facilitating lexical activation, segmentation of the speech stream utilizing lexicallydriven strategies is promoted.

**Perceptual Learning**

The notion of training someone to be a better listener is often met with the criticism of feasibility. Why would training focus on a single listener when a speaker will talk with many people? However, perceptual training appears to be a viable intervention strategy when an individual has a limited number of caregivers and little ability or inclination to modify the quality of their speech output. An emerging body of evidence shows not only the ability to improve a listener's understanding of hypokinetic dysarthria but also sustained improvement over time [83].

Varied exposure to degraded speech has shown benefits to subsequent processing. This has been demonstrated for foreign-accented speech [84–86], noise-vocoded speech [32, 87], time-compressed speech [88], and, critically, for dysarthric speech [31, 89]. Liss and her colleagues [31] briefly exposed listeners to speakers diagnosed with either hypokinetic or ataxic dysarthria prior to a transcription task. Relative to a control condition that provided no training, listeners enjoyed perceptual benefits of previous exposure as measured by increased percent words correct on the transcription task. The length of training and type of feedback are also factors that impact the magnitude of improvements in intelligibility [83]. Additionally, research in perceptual training may expand beyond that of auditory training to include the use of visual information (i.e., training using video samples) to expand the listener's understanding of how the speaker's articulatory movements match with their vocal output.

Currently, the precise mechanisms responsible for increased intelligibility following prior exposure are being explored. Preliminary evidence suggests that learning can and does occur at the phonemic level in that mental representations are able to better accommodate the degraded acoustic-phonetic information following exposure [31, 83]. Relatively stable speech features, even if impaired relative to control, have the potential to be robust cues to lexical segmentation and activation under the paradigm of perceptual training. Perceptual training with degraded speech allows listeners to exploit such regularities to become more adept at identifying word boundaries and activating the intended words. Specifically, intervention exploiting the diminished but still present cues to syllabic stress in speakers with hypokinetic dysarthria, be it training the listener or improving the quality of the speech signal (e.g., via prosodic exercises), is justified by this cognitive-perceptual approach.

## 4. Conclusions and Recommendations

The approach proposed herein is intended to augment the clinical decision-making process in the referral and treatment of PD patients with hypokinetic dysarthria. As the evidence base grows, decisions about treatment targets and intervention strategies can be motivated by both their intended impact on speech output, as well as strategies employed by listeners. Rate reduction techniques that target the speech features of hypokinetic dysarthria most closely associated with articulation (e.g., rapid, imprecise, short rushes of speech, dysfluency) have the potential to facilitate articulatory precision and afford the listener increased processing time to complete the tasks of lexical segmentation and activation. Speech treatment techniques that elicit louder speech (e.g., LSVT) not only aim to remediate audibility of the acoustic-phonetic information but also aim to improve the quality of stress cues that have been demonstrated to facilitate lexical segmentation (e.g., pitch and loudness variations). 

However, the theoretical underpinnings of the aforementioned interventions may better inform treatment decisions. As there is little empirical information upon which clinicians can base their strategic decisions, future research must focus on how different forms of signal-complementary information (e.g., cues, training methods) can facilitate different avenues for improving intelligibility. Once the mechanisms of remediation are better understood, it may be possible to combine different types of signal-complementary information with one another and other therapeutic techniques with predictable outcomes. From this perspective, future well-designed investigations of treatment efficacy may compare mechanisms of intelligibility gains in this population in ways that converge on a gold standard intervention regimen.
